# A rapid immunochromatographic test for antibody detection as a new tool for monitoring malaria caused by *Plasmodium vivax*

**DOI:** 10.1371/journal.pntd.0014447

**Published:** 2026-06-26

**Authors:** Julia Ramos Sampaio, Fabiana Fioravante Coelho, Irene da Silva Soares, Jéssica Karoline Augusta Oliveira, Ana Clara Gazzinelli-Guimarães, Alex Fiorini Carvalho, Bruno Vinicius Santos Valiate, Camila Medeiros Costa, Caroline Junqueira, Alexia Martines Vieira Silva, Michelle Oliveira-Silva, Dhelio Batista Pereira, Anielle de Pina-Costa, André Machado de Siqueira, Marcia Caldas de Castro, Renan Pedra de Souza, Lis Ribeiro do Valle Antonelli, Ana Paula Salles Moura Fernandes, Ricardo Tostes Gazzinelli, Gregório Guilherme Almeida

**Affiliations:** 1 Vaccine Technology Center, Federal University of Minas Gerais, Belo Horizonte, Minas Gerais, Brazil; 2 School of Pharmaceutical Sciences, University of São Paulo, São Paulo, Brazil; 3 René Rachou Research Institute, Oswaldo Cruz Foundation, Belo Horizonte, Minas Gerais, Brazil; 4 Tropical Medicine Research Center, Porto Velho, Rondônia, Brazil; 5 Fluminense Federal University, Rio de Janeiro. Brazil; 6 Evandro Chagas National Institute of Infectious Diseases, Oswaldo Cruz Foundation, Rio de Janeiro, Brazil; 7 Department of Global Health and Population, Harvard T.H. Chan School of Public Health, Boston, Massachusetts, United States of America; AstraZeneca plc, UNITED STATES OF AMERICA

## Abstract

Eliminating malaria caused by *Plasmodium vivax* remains a challenge, especially in areas of low transmission, where the high proportion of asymptomatic and subpatent infections limits the effectiveness of national eradication program control strategies. Antibody levels against parasite’s antigens wane several months after treatment. Since the acquisition and maintenance of antimalarial humoral immunity depends on the infection, antibodies can serve as biomarkers of parasite exposure, emerging as a promising alternative for monitoring recent exposure to the parasite. We developed and validated a rapid diagnostic test that detects IgG (Ab-RDT) against two blood-stage antigens of *P. vivax*. The Ab-RDT identified a gradual decrease in antibody levels in treated individuals, suggesting that a positive test reflects recent exposure to the parasite or maintenance of infection. In agreement, a longitudinal study showed that a positive test, on Ab-RDT or ELISA, was strongly associated with the risk of subsequent detection of parasitemia by qPCR, highlighting the tests’ usefulness in identifying individuals who were recently exposed or are more likely to be infected. The field performance of the Ab-RDT was 80.1% sensitivity and 89.4% specificity, using ELISA as a reference method, reinforcing the potential of antibody detection as a valuable tool to support malaria surveillance for *P. vivax*, especially for monitoring transmission in regions moving towards eradication, thereby enabling more strategic efforts in malaria control.

## Author summary

Malaria caused by *Plasmodium vivax* may lead to very low parasitemia, limiting detection of infection, especially in asymptomatic individuals. Since these silent infections can maintain transmission, the lack of diagnosis and effective treatment hinders the effectiveness of malaria eradication program interventions. Antibodies produced against some antigens reflect recent exposure to the parasite and are more stable markers than parasite detection by blood smear, antigen-RDT or PCR. Antibody-RDTs can serve as useful indicators of transmission, offering an alternative to the limitations of current tests. We developed a rapid diagnostic test to detect antibodies (Ab-RDT) against blood-stage antigens of *P. vivax*. The prototype showed high sensitivity and specificity under controlled conditions and was validated in field missions. Antibody levels decreased after treatment, indicating that positive results are associated with recent exposure. Among residents of an endemic area over time, a positive test result was associated with higher risk of detecting parasite DNA later. Besides its several practical advantages for accessible diagnosis in field conditions, the Ab-RDT would allow the screening of people who are exposed, but do not show symptoms or are missed by parasitological or molecular diagnostic methods, serving as potential carriers that can favors transmission. The test might be useful in the control strategies by enabling rapid and cost-effective monitoring of exposure, guiding targeted interventions, and monitoring the impact of control measures.

## Introduction

Malaria remains one of the most significant global public health challenges, with millions of cases reported annually and persistent endemic transmission in several regions worldwide [1]. In Brazil, despite significant progress achieved in recent decades, the disease continues to prevail in the Amazon region, where *Plasmodium vivax* accounts for approximately 80% of reported infections [1,2].

One of the main obstacles to *P. vivax* control is the high proportion of asymptomatic individuals, which may account for up to 90% of infections [3]. These carriers do not seek medical care and remain a source of transmission, as they often harbor submicroscopic parasitemia, which is undetectable by conventional diagnostic methods such as light microscopy and rapid antigen tests. Even molecular assays may yield false-negative results when blood sampling occurs during periods of very low or undetectable circulating parasitemia [4–6]. The early presence and rapid maturation of gametocytes in this infection profile further increase the potential for silent dissemination of the parasite, representing a critical barrier to elimination efforts [7].

The Brazilian Ministry of Health launched the National Malaria Elimination Plan in 2022, which aims to interrupt transmission by 2035, in alignment with the Global Technical Strategy for malaria. Within this framework, diagnosis plays a central role, not only in detecting and treating active cases but also in mapping residual transmission foci, guiding targeted interventions, and monitoring the impact of control measures [1,8]. Asymptomatic individuals harbor a very small parasitemia, which can be missed by molecular methods. In fact, parasitemia in these individuals oscillate at least weekly imposing a challenge for the diagnosis [5]. On the other hand, serum titers against parasites antigens are a more stable biomarker, lasting week to months to fall below the detection levels. In this sense, antibody detection using *P. vivax* antigens serves as a complementary surveillance strategy, as they can be used as biomarkers of both transmission intensity and recent exposure. Serological approaches, such as serological monitoring, can be useful in estimating disease burden, identifying potential asymptomatic individuals or those at risk of infection, supporting control strategies, and optimizing health resource allocation. [9–12].

Although serological assays such as ELISA are valuable tools for detecting previous exposure to *P. vivax*, their application in remote endemic settings is limited by the need for laboratory infrastructure, specialized equipment, and trained personnel. In contrast, currently available malaria rapid diagnostic tests (RDTs) are primarily designed for antigen detection and have reduced sensitivity in low-parasitemia and asymptomatic infections. Therefore, there is a lack of field-deployable serological tools capable of rapidly identifying exposed individuals in endemic areas.

We herein present a novel lateral flow rapid diagnostic test for detecting antibodies against blood-stage antigens of *P. vivax* (Ab-RDT). We hypothesize that the Ab-RDT can be used as a field-applicable point-of-care alternative to support epidemiological surveillance activities, with practical advantages compared to ELISA and qPCR that requires specialized equipment, reagents and trained personnel. Two key immunogenic targets were selected: the 19-kDa C-terminal fragment of the merozoite surface protein 1 (*Pv*MSP1₁₉) and the apical membrane antigen 1 (*Pv*AMA-1). *Pv*MSP1₁₉ is characterized by inducing predominantly short-lived antibody responses after antimalarial treatment and has been associated with the detection of recent exposure to *Plasmodium vivax*. In addition, *Pv*MSP1₁₉ has been extensively evaluated serologically in other studies, showing low cross-reactivity with samples from African individuals with no history of *P. vivax* infection [13–16]. *Pv*AMA-1 is recognized as a sensitive biomarker of recent exposure in endemic areas, particularly in low-transmission settings, in which the frequency of specific IgG antibody responses varies according to the number of previous infections. Both antigens have been extensively evaluated in serological studies using samples from different regions of Brazil, reinforcing their applicability and epidemiological relevance for the development of the rapid test [17–20].

## Methods

### Ethical approvals

This study was performed under protocols reviewed and approved by the Ethical Committees on Human Experimentation from Instituto René Rachou, Centro de Pesquisas em Medicina Tropical de Rondônia, and National Ethical Council (CAAE: 59902816.7.0000.5091 – standardization panel, 43253921.7.0000.5091 – cross-sectional and 5-point longitudinal study and 524757.21.2.1001.5262 – ABRACAMAL). All participants were informed about the objectives and procedures of the study, with voluntary participation through written informed consent.

### Biological samples

We deployed two major sample panels in this study. The first was the sample panel for standardizing the Ab-RDT (standardization panel) which consisted of plasma from healthy donors (CTL, n = 20) with no previous record of malaria, and symptomatic (SY, n = 30) and asymptomatic individuals (ASY, n = 49), both characterized by positive qPCR for *P. vivax* with or without common malaria symptoms, respectively. Both groups (SY and ASY) showed indirect ELISA positivity [5]. Samples were collected between 2018 and 2019, in Candeias do Jamari and Porto Velho, Brazil. Healthy donors were from Belo Horizonte, Minas Gerais, a non-endemic area for malaria.

The second panel (validation panel) comprised samples from two endemic areas (n = 449; total of 1,299 samples), comprising whole blood collected by either fingerprick or venipuncture in EDTA anticoagulant. These samples came from three field studies: (1) a 1-year longitudinal study with treated symptomatic individuals enrolled in the ABRACAMAL project (longitudinal study, n = 34; total of 176 samples; Porto Velho, Rondônia, Brazil, 2023); (2) single-point collection of asymptomatic individuals (cross-sectional study, n = 221; Candeias do Jamari, Rondônia, Brazil, 2023); (3) 5-points longitudinal study with residents with unknown serological or parasitological status (longitudinal study, n = 192; total of 902 samples; Candeias do Jamari, Rondônia, Brazil, 2025). In all studies, the inclusion criteria comprised only adult individuals (18–65 years old), non-pregnant and non-lactating, with no cognitive impairment. Participants who declined to participate after providing informed consent were excluded from the analyses. Because this study used previously established and available serum panels, a formal prospective sample size calculation was not performed.

### Antigens

The recombinant antigens were provided by Dr. Irene Soares from the Department of Clinical and Toxicological Analysis of the School of Pharmaceutical Sciences of the University of São Paulo. *Pv*AMA1, corresponding to the ectodomain region (amino acids 43–487) derived from a Brazilian clinical isolate of *P. vivax*, was expressed in *Pichia pastoris*. The expression and purification procedures have been previously described [21]. *Pv*MSP1_19_, corresponding to the 19-kDa C-terminal fragment of *P. vivax* MSP1 (Belém strain; amino acids 1616–1704), was expressed in *Escherichia coli* as previously reported [22]*.* Both antigens were purified by affinity chromatography followed by ionic exchange chromatography. All proteins were characterized by the Quality Control Facility of CT Vacinas, prior to use, to confirm protein identity, integrity, and purity. In addition, Ab-RDT production was conducted under strict quality control procedures, with all lots manufactured in accordance with Good Laboratory Practice (GLP) standards.

### Molecular diagnosis

DNA extraction from whole blood was performed according to the manufacturer’s protocol for the MagMAX DNA Multi-Sample Ultra 2.0 kit, using the KingFisher Apex plate (Thermo Fisher Scientific). After extraction, a qPCR targeting a sub-telomeric non-ribosomal multicopy target (Pvr47) was performed as described previously [5,23]. Briefly, the reaction mix consisted of 0.5 μM primers (Pvr47-Forward 5’-TCCGCAGCTCACAAATGTTC3–3’, Pvr47-Reverse 5’-ACATGGGGATTCTAAGCCAATTTA-3’), 0.25 μM probe (fam-5’-TCCGCGAGGGCTGCAA-3’-MGB), 5 μL reaction buffer (CTVacinas, Brazil), 0.5 μL Taq polymerase, and 0.5 μL nuclease-free water. The plates containing this mix were lyophilized using the Epsilon 2-6D LSCplus equipment (Martin Christ Freeze Dryers) according to the following protocol: 96-well plates (Sarstedt) were loaded into the equipment and cooled to -40 °C for 30 minutes. The plates were then lyophilized at a vacuum of 0.2 mbar for 24 hours. After the overnight process, a vacuum of 1 µbar was applied for 2 hours. Then, the vacuum was removed, and the plates were hermetically sealed in plastic packets (NZB Embalagem) at 4 °C until qPCR was performed. For the reactions, 10 µL of DNA extracted from the participants’ blood was added in triplicate, as well as a standard curve, blanks, positive, and negative controls. The reactions were performed with the following steps: 10 minutes of initial denaturation at 95 °C, followed by 45 cycles of denaturation (95 °C/3s), annealing (54 °C/30s), and extension (60 °C/30s). A 6-point standard curve (10^5^ to 1 copies/μL) was constructed using a TOPO-2.1 plasmid with the amplified fragment inserted to calculate the copy number of the target in a sample. The positive control consisted of a sample with a Quantification Cycle (*Cq*) of 20, used to calibrate and compare across different batches. The blank consisted of 10 µL of PCR-grade water, and the negative control consisted of a DNA sample from a participant from a non-endemic area. We consider an infected individual as one with at least one positive qPCR result in a triplicate.

### IgG detection by indirect ELISA

The indirect ELISA protocol was performed as described by Figueiredo et al. [24] and Almeida et al. [5], with the following modifications: the recombinant proteins *Pv*MSP1₁₉ and *Pv*AMA-1 were co-immobilized on high-binding polystyrene plates (Thermo Scientific) at concentrations of 20 ng/well and 100 ng/well, respectively, diluted in carbonate–bicarbonate buffer and stored overnight at 4 °C. Plates were then incubated for 2 hours with a blocking solution containing 2% (w/v) bovine serum albumin (BSA). The samples were diluted 1:100 in sample diluent containing 1% (w/v) BSA and incubated at 37 °C for 45 minutes. Plates were washed five times with 300 µL of PBS-Tween 0.1% using an automatic plate washer (Thermo Scientific), and residual liquid was removed by manual inversion onto absorbent paper. Subsequently, horseradish peroxidase (HRP)-conjugated anti-human IgG (Fc-specific) antibody was added to each well, and plates were incubated at 37 °C for 30 minutes. After another washing step with PBS-Tween 0.1%, reactions were developed by adding TMB substrate (3,3’,5,5’-tetramethylbenzidine, Sigma) and incubating for 30 minutes at room temperature. The reaction was stopped by adding 0.5 M sulfuric acid (H₂SO₄) stop solution.

### Antibody rapid diagnostic test (Ab-RDT)

**Fig 1:**
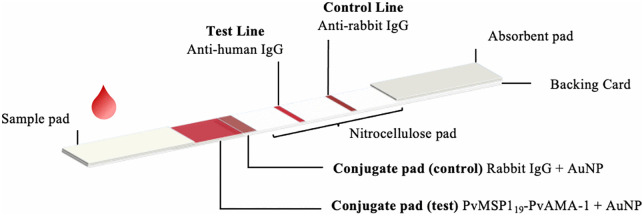
Schematic of the proposed Ab-RDT format. The test and control lines contain anti-human IgG and anti-rabbit IgG antibodies, respectively, impregnated on the nitrocellulose pad. The test and control conjugate pads contain *P. vivax* proteins and anti-rabbit IgG antibodies conjugated to colloidal gold nanoparticles, respectively. The sample pad can be used with blood or plasma.

**Fig 2:**
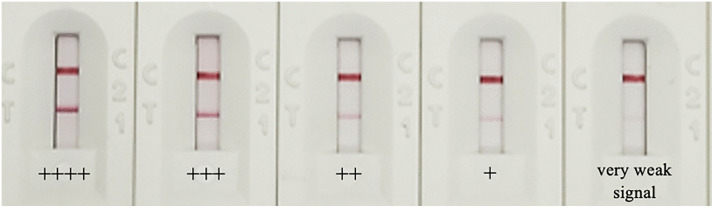
Representation of the signal intensity of the test line in the Ab-RDT. Representative figure of Ab-RDT results by category. According to the signal intensity of the positive samples on the test line (LT), the Ab-RDT results were qualitatively categorized into four crosses (++++, high intensity, very dark red color), three crosses (+++, moderate to high intensity, dark red color), two crosses (++, moderate do low intensity, red color), one cross (+, low intensity, light red color), and into a very weak signal (very light red color).

The antibody rapid diagnostic test consisted of a test line (TL) containing a mouse anti-human IgG (Fapon) and a control line (CL) containing goat anti-rabbit IgG (Sigma), both immobilized on a nitrocellulose pad (0.5–4.0 mg/mL). The system also included two conjugate pads: one containing the two *P. vivax* proteins conjugated to colloidal gold nanoparticles (AuNP, the test conjugate), and the other containing rabbit IgG conjugated to AuNP (the control conjugate, Fig 1). The AuNP production followed the protocol of Frens [25]. A test result was considered positive when any visible signal was observed in the test line in conjunction with the control line, whereas negative results were defined by the presence of a signal exclusively in the control line. Tests lacking a visible control line were considered invalid, discarded, and repeated using a new device. For positive samples, the intensity of the color signal in the test line was qualitatively categorized into four crosses (++++, high intensity, very dark red color), three crosses (+++, moderate to high intensity, dark red color), two crosses (++, moderate do low intensity, red color), one cross (+, low intensity, light red color), and into a very weak signal (very light red color) (Fig 2).

### Validation phase

The validation process was conducted in three stages: (1) Assessed 34 symptomatic individuals treated with primaquine who were followed for one year with no reported relapse or reinfection. Between 4 and 5 post-treatment sampling points were analyzed per participant, starting on day 28 after treatment initiation. At each point, plasma samples were tested by both ELISA and the Ab-RDT to monitor the persistence and decline of antibody responses. (2) The Ab-RDT test was performed once per participant on 221 individuals from Candeias do Jamari, using plasma collected from whole blood obtained by finger prick directly at the participants’ homes. (3) Consisted of a study involving 192 residents of Candeias do Jamari, tested once a week for 5 weeks. Each participant enrolled in stages 2 and 3 was tested using the rapid test, quantitative PCR (qPCR), and indirect ELISA.

### Statistical analysis

The cut-off values for the ELISA were defined based on the samples from 20 healthy donors with no previous malaria included in the standardization panel. The cut-off was the arithmetic mean of the optical density readings from these samples, plus three standard deviations from each assay. The results were expressed as a reactivity index (relative index; RI), according to the following formula:

*RI = Sample optical density/ Cut-off*

Each sample was classified as positive or negative for the antibody. Samples with relative index values below 0.9 were considered negative; those above 1.1 were considered positive; and samples with relative index values between 0.9 and 1.1 were considered indeterminate. ELISA was selected as the reference method because both assays are based on serological detection and provide more consistent measures of exposure than PCR in the context of low-density and fluctuating asymptomatic infections. Sensitivity, specificity, and accuracy were calculated according to the following formulas [26], where TP = true positives; TN = true negatives; FP = false positives; FN = false negatives:

*Sensitivity = (TP/ (TP + FN))*
*Specificity = (TN/ (TN + FP))*
*Accuracy = (TP + TN)/ total number of samples tested*

The 95% confidence intervals (CIs) for sensitivity, specificity, and accuracy were calculated using the Wald and Clopper-Pearson methods. The Mann-Whitney and Fisher tests were used to compare means between groups, and differences were considered statistically significant when p < 0.05. Agreement between tests was calculated using Cohen’s Kappa index (κ) [27] and interpreted according to Landis and Koch [28]: 1.00–0.81 = excellent agreement; 0.80–0.61 = good; 0.60–0.41 = moderate; 0.40–0.21 = fair; and 0.20–0.00 = slight agreement.

The decay over time was modeled using a nonlinear Bayesian model fitted with the *brms* package, assuming a Gamma distribution with a log link. The conditional mean was specified as μ(t) = A exp(−k t), with A > 0 and k > 0 ensured through log parameterization (i.e., A = exp(logA) and k = exp(logk)). Weakly informative priors were used for the nonlinear parameters (logA ~ N(0, 2), logk ~ N(−1, 1)), and an exponential prior was specified for the Gamma shape parameter. Posterior inference was obtained via Hamiltonian Monte Carlo sampling (NUTS) with four chains and 4,000 iterations per chain, with adaptation controls. Model fit was assessed using NUTS diagnostics, posterior predictive checks comparing simulated and observed distributions, and summary statistics, including posterior predictive checks conditional on time. The population half-life was derived from the posterior distribution of k as t₁⁄₂ = ln(2)/k, reporting the median and the 95% credible interval (CrI). The probability of being infected in the previous nine months given that a patient has a positive ELISA test was estimated using posterior samples from a Bayesian nonlinear Gamma regression model fitted in brms. Time-to-event analyses were performed in R using the *survival* and *survminer* packages to assess the association between baseline diagnostic status and the occurrence of PCR positivity during follow-up. The event was defined as the first PCR-positive result, and time was measured as the study visit at which PCR positivity was first detected. Participants without PCR positivity during follow-up were right censored at the last visit. Survival analyses were conducted separately according to baseline rapid test and ELISA status. ELISA positivity was defined using a predefined threshold (RI > 1.1). Group status was treated as a fixed covariate. Cumulative incidence curves were estimated using the Kaplan–Meier method, and associations were quantified using Cox proportional hazards models, from which hazard ratios (HRs) and 95% confidence intervals (CIs) were obtained, with statistical significance set at p < 0.05.

Data tables were compiled in Excel spreadsheets, and formal analyses were performed in GraphPad Prism (version 8) or R (version 4.5.0).

## Results

### Rapid diagnostic test development

**Fig 3:**
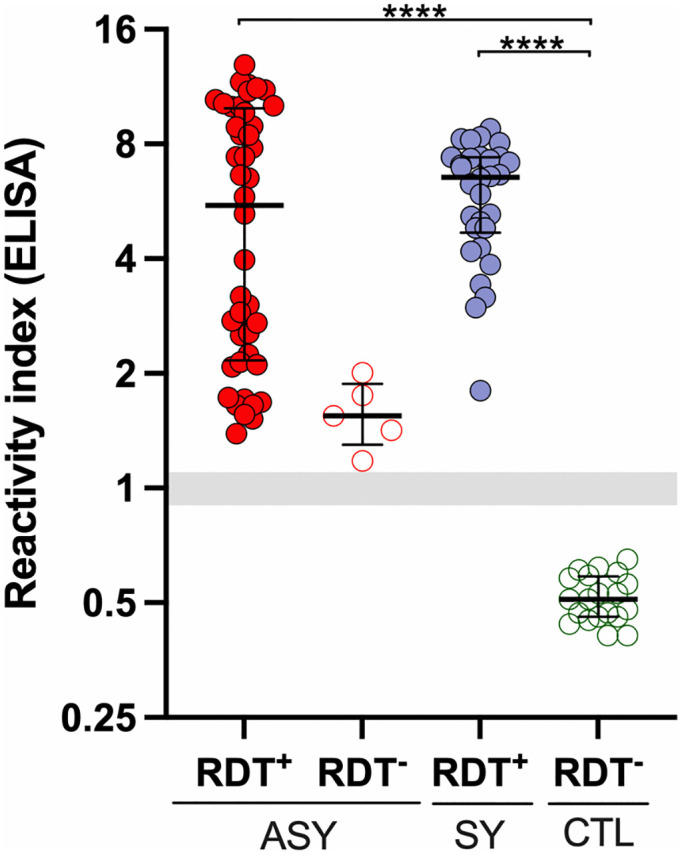
Evaluation of the Ab-RDT using the standardization panel. The panel included plasma samples from asymptomatic individuals (ASY; orange), symptomatic individuals (SY; purple), and healthy donors from a non-endemic area (CTL; green). Each dot represents a participant. Filled dots represent positive Ab-RDT results (RDT+), while open dots represent negative results (RDT-). The gray line indicates the ELISA reactivity index cut-off (positive >1.1; negative <0.9). Bars represent median and interquartile range, with the y-axis shown on a Log₂ scale. Dunns test, ****p < 0.0001.

Several prototypes were tested with the standardization panel until we achieved sensitivity and specificity higher than 90%. The final Ab-RDT achieved a sensitivity of 93.7% (95% CI: 88.3–99.0%) and a specificity of 100% (95% CI: 83.1–100%), compared to ELISA when used standardization panel (Fig 3). The Ab-RDT showed an excellent performance in identifying symptomatic (30/30, 100%) and asymptomatic (44/49, 89.8%) individuals, from the standardization panel. This prototype reached an accuracy of 94.9% (95% CI: 90.6–99.2%) and demonstrated an excellent agreement with the ELISA assay (κ = 0.86) (Table 1).

**Table 1:** Performance of the Ab-RDT using the standardization panel compared to ELISA results.

Parameters	Value (detected/n) (95% CI)
**Sensitivity**	93.7% (74/79) (88.3% - 99.0%)
**Specificity**	100% (20/20) (83.2% - 100.0%)
**Accuracy**	94.9% (90.6% - 99.3%)
***Kappa* index**	0.86 (excellent)

### Antibody kinetics in treated symptomatic individuals

An antibody-based RDT will measure IgG presence and thus indirectly detect a previous infection. It is known that IgG levels against the tested antigens drop rapidly after treatment [13,18]. Therefore, we aimed to evaluate the meantime the treated participants became serologically negative in both ELISA and Ab-RDT. A total of 34 plasma samples from the ABRACAMAL project were analyzed from individuals with symptomatic *P. vivax* malaria who were diagnosed and treated in Porto Velho, Rondônia. Samples from four to five collection points were used, starting 28 days after treatment. Only those who did not present relapse or reinfection (positive in microscopy) during the follow-up were included in this analysis.

As shown in Table 2, the proportion of individuals exhibiting strong signal intensity (3 or 4 crosses) in the Ab-RDT was higher within the first six months after treatment. A gradual decrease in signal intensity and/or test negativity was observed thereafter, reaching the lowest levels at 12 months post-treatment, although the number of samples available at later time points was limited. These findings were consistent with the ELISA reactivity index (RI), which showed higher values during the first six months post-treatment, followed by a marked decline in subsequent months.

**Table 2:** Signal intensity in the Ab-RDT according to post-treatment follow-up times.

Post-treatment sampling time (days)	4 crosses (n)	3 crosses (n)	2 crosses (n)	1 cross (n)	Very weak signal (n)	Negative (n)	Median (ELISA RI)
28 (n = 34)	88.2% (30)	5.9% (2)	5.9% (2)	–	–	–	5.69
42 (n = 31)	90.3% (28)	–	3.2% (1)	6.4% (2)	–	–	5.43
60 (n = 33)	78.8% (26)	6.1% (2)	6.1% (2)	3.0% (1)	3.0% (1)	3.0% (1)	4.14
120 (n = 32)	65.6% (21)	3.1% (1)	6.2% (2)	9.4% (3)	9.4% (3)	6.2% (2)	2.58
180 (n = 31)	48.4% (15)	6.4% (2)	6.4% (2)	16.1% (5)	12.9% (4)	9.7% (3)	1.79
270 (n = 10)	10.0% (1)	10.0% (1)	–	30.0% (3)	10.0% (1)	40.0% (4)	1.11
360 (n = 5)	40.0% (2)	–	–	–	20.0% (1)	40.070% (2)	0.56

(-) = No samples from the indicated period showed that intensity.

**Fig 4:**
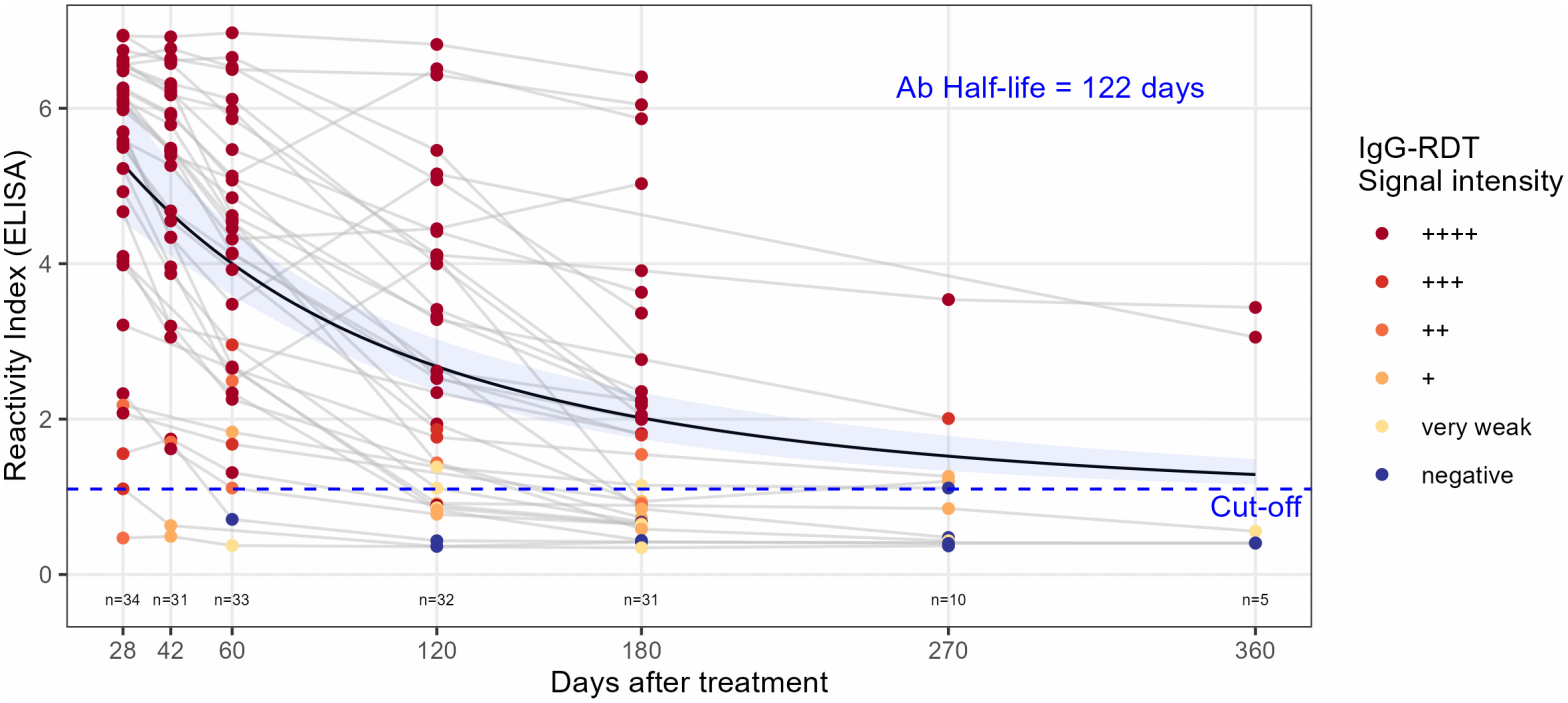
IgG levels and RDT signal intensity over time among treated *Plasmodium vivax* patients without reinfection. Thirty-four patients with *P. vivax* malaria were treated and followed-up for one year (x-axis). The y-axis represents the ELISA reactivity index, and the dashed blue line indicates the ELISA cut-off (positive >1.1; negative <0.9). Each patient is represented by a dot, and paired measurements are connected by the gray lines. Dots were colored to represent the Ab-RDT signal intensity at each time point. The black curve represents the model fit median with 95% credible interval.

A subset of participants (n = 4) exhibited transient increases in ELISA reactivity between 60 and 180 days post-treatment, possibly due to a reinfection. However, this hypothesis could not be confirmed due to the lack of parasitological or molecular follow-up during that interval. Antibody levels to the *Pv*MSP1₁₉ and *Pv*AMA-1 antigens approached the Ab-RDT detection threshold approximately 270 days after treatment. Based on these findings, the estimated half-life of the antibodies detected by the ELISA was 122 days (CrI: 93.4 – 165.0). When positive, the ELISA indicated a 96.0% (86.9 – 99.5% CrI) probability that the sample was collected within nine months after infection (Fig 4).

### Longitudinal analysis with asymptomatic individuals

**Fig 5:**
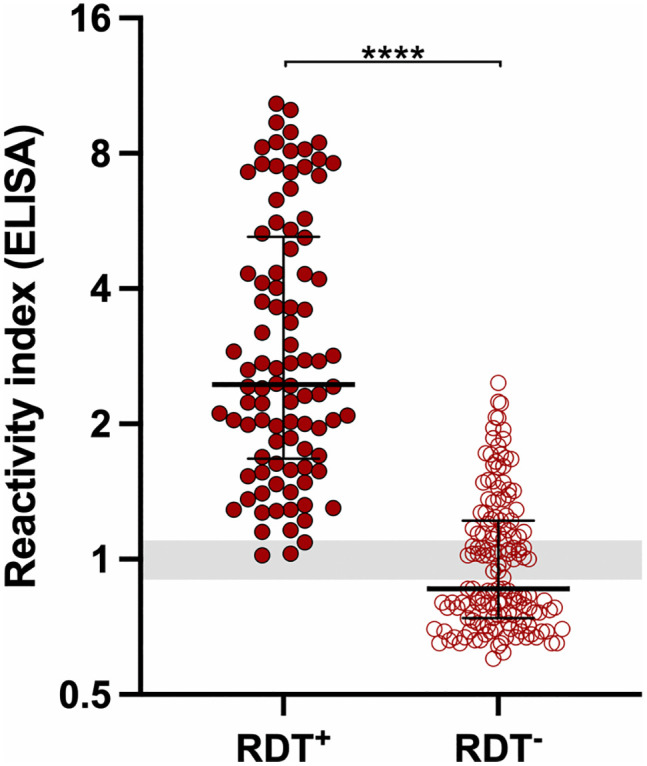
Evaluation of the Ab-RDT using the sample panel from stage 2 field validation. Each dot represents a participant. Filled red dots represent positive samples (RDT + , n = 94), and open circles represent negative samples (RDT-, n = 155). The y-axis represents the antibody levels in the ELISA and is shown on a Log₂ scale. The gray line indicates the ELISA cut-off range (positive >1.1; negative <0.9). Vertical bars and whiskers represent the median and interquartile range. Mann-Whitney test, ****p < 0.0001.

In the second stage of field validation, a single collection of 221 plasma samples was carried out from exposed participants living in Candeias do Jamari (Rondônia, Brazil). No individual was positive by microscopy, while 13 were positive by qPCR. Among qPCR-positive participants, only 8 (8/221, 3.6%) were positive in Ab-RDT or ELISA. ELISA and Ab-RDT showed moderate agreement (κ = 0.55). Importantly, the Ab-RDT identified samples with low ELISA reactivity indexes, showing greater sensitivity within the borderline range of serological responses. In addition, the test showed good discrimination among negative samples, sustaining the high specificity. Fig 5 illustrates the relationship between the ELISA reactivity index and the Ab-RDT results.

To overcome the limitations of identifying asymptomatic *P. vivax* infections by microscopy or molecular methods when using a single-sample collection, a five-week longitudinal study (stage 3) was conducted with 192 residents of Candeias do Jamari. Among them, 161 individuals completed all scheduled collections. Weekly blood samples were analyzed by Ab-RDT, ELISA, and qPCR, enabling the assessment of recent exposure dynamics and agreement among the methods. Since previous studies failed to detect asymptomatic infections by microscopy, we did not include this method in this study.

**Fig 6:**
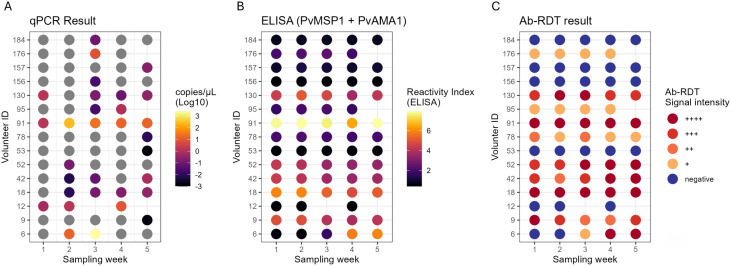
Longitudinal *Plasmodium vivax* detection and antibody responses among participants of Candeias do Jamari, Brazil. Representative dot-heatmaps of 15 participants that displayed at least one positive qPCR for *P. vivax*. Each dot in the horizontal line represents one participant. Missing data is represented as empty positions at the plot. The three plots are depicting: (A) qPCR results in copies/µL over five consecutive weeks. Color intensity indicates parasite density on a Log₁₀ scale. Gray circles indicate undetectable parasite DNA. **(B)** IgG antibody responses by indirect ELISA using the combined PvMSP1₁₉ and PvAMA-1 antigens, with reactivity index color-coded by reactivity index. **(C)** Ab-RDT signal intensity by week and color-coded by the qualitative result: negative (blue) or positive at increasing intensities: + (yellow), ++ (orange), +++ (red), and ++++ (dark red).

*P. vivax* DNA was detected in 15 participants (15/192, 7.8%), 12 of whom completed all collections (12/161, 7.4%). Among these, eight (IDs 9, 52, 53, 78, 156, 157, 176, and 184) were positive in only one week, two (IDs 12 and 91) remained positive throughout the study, and the others alternated between positive and negative results. One individual (ID 6) presented clinical malaria (fever, malaise, chills, muscle pain, and headache) in week 3, although we could only detect *P. vivax* DNA in week 2. The infection was confirmed by microscopy at week 3 in the local health service, and the participant received standard treatment. Despite this, all other infections were classified as asymptomatic (Fig 6A).

Among asymptomatic PCR-positive individuals, five (IDs 12, 53, 156, 157, and 184) were seronegative by ELISA or Ab-RDT (5/14, 35.7%) and were positive in only one replicate, except for ID 12 (positive in all replicates), with *Cq*s < 35. Three (IDs 53, 130, and 184) reported never having had malaria; three (IDs 18, 91, and 95) had infections less than one year prior, and the remaining individuals reported previous infections over two years earlier (Fig 6B). As observed in Fig 3, the Ab-RDT signal intensity closely paralleled the ELISA reactivity index (Fig 6C).

Among the 192 participants, 74–86 tested positive by ELISA each week, of whom approximately 61–68 were also detected by Ab-RDT. Weekly sensitivity ranged from 70.9% to 84.2%, while specificity varied between 89.4% and 93.6% (Table 3).

**Table 3:** Weekly sensitivity and specificity of Ab-RDT compared to ELISA during longitudinal follow-up.

Parameters	Week 1	Week 2	Week 3	Week 4	Week 5
Sensitivity (95% CI)	79.1% (69.3% –86.3%) (68/86)	71.0% (60.5%– 79.6%) (61/86)	77.4% (67.4%– 85.0%) (65/84)	83.8% (74.2%– 90.2%) (62/74)	84.2% (74.8%– 90.5%) (64/76)
Specificity (95% CI)	90.6% (82.5%–95.3%) (8/85)	89.4% (79.4%– 95.0%) (7/66)	93.5% (84.3%– 97.4%) (4/62)	90.0% (80.6%– 95.1%) (7/70)	93.2% (85.0%– 97.1%) (5/74)

Values represent the number of Ab-RDT-positive samples among ELISA-positive samples per week.

The sensitivity calculation used samples that were positive in the ELISA test, and the specificity calculation used samples that were negative in the ELISA test. Numbers in between parentheses represent the number of samples detected in the Ab-RDT/ total number of samples in the ELISA per week.

**Fig 7:**
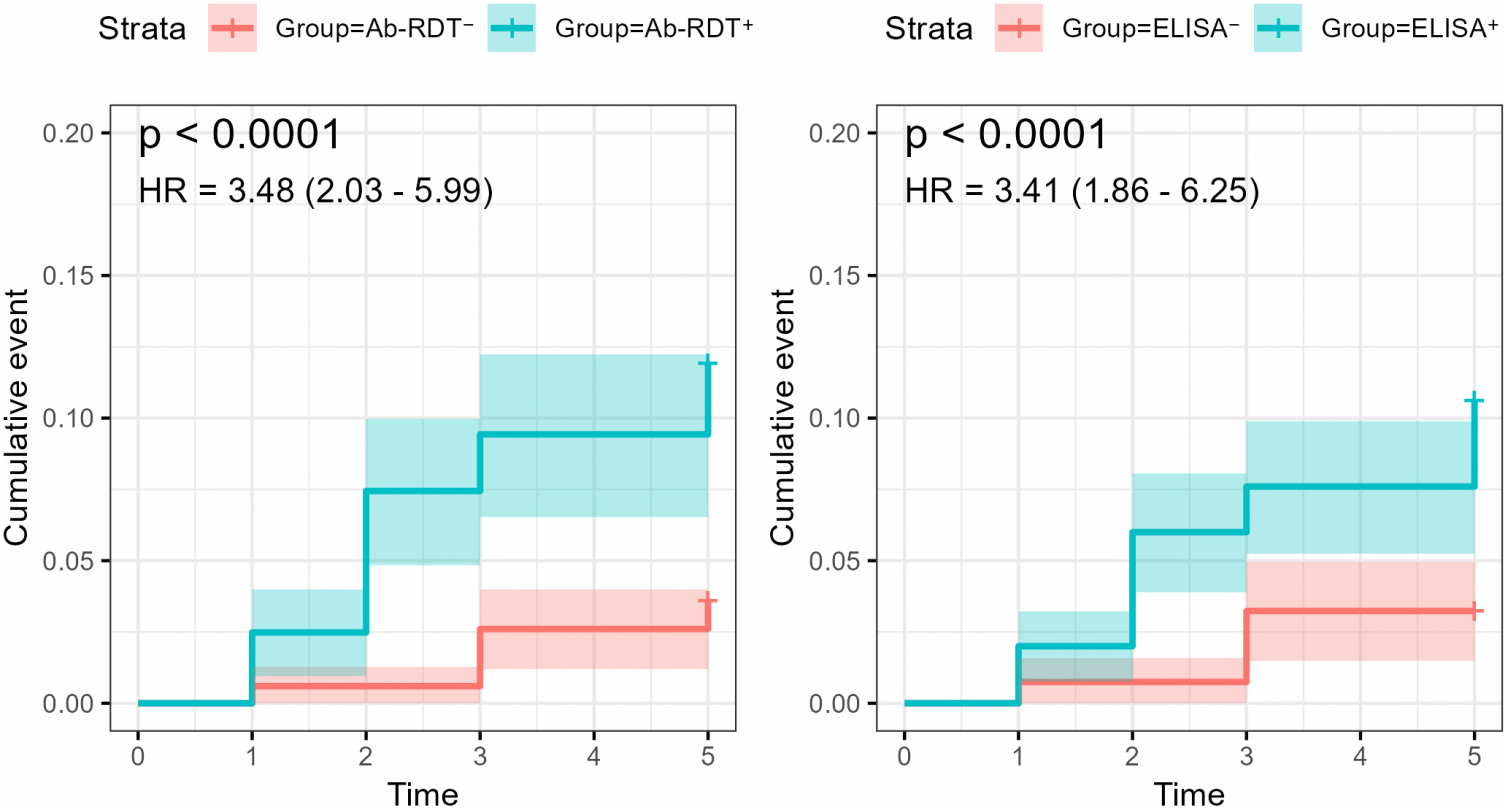
Cumulative incidence of PCR positivity according to baseline diagnostic status. Kaplan–Meier cumulative event curves showing the time to first qPCR-positive result stratified by baseline rapid test result (Ab-RDT) status (left) and baseline ELISA status (right). Time in weeks is represented in x-axis. Participants without qPCR positivity during follow-up were right censored at the last visit. Hazard ratios (HRs) and 95% confidence intervals (CIs) were estimated using Cox proportional hazards models.

Risk analysis showed that positivity in both ELISA and Ab-RDT was significantly associated with subsequent detection of parasitemia by qPCR. Individuals who were positive by ELISA had a hazard ratio (HR) of 3.41 (95% CI: 1.86–6.25; p < 0.0001) for qPCR positivity in subsequent weeks, while those positive by Ab-RDT showed an even higher HR of 3.48 (95% CI: 2.03–5.99; p < 0.0001) (Fig 7). These results highlight the ability of both tests to identify individuals who have been recently exposed to *P. vivax* and are at increased risk of developing a detectable infection, even in the absence of clinical symptoms.

### Overall performance of the Antibody Rapid Test

Since the aim of this study was to develop an antibody-based RDT with performance comparable to ELISA for field applications, ELISA was used as the reference standard. Considering all samples from Rondônia, the overall sensitivity of the Ab-RDT was 80.1% (95% CI: 76.9–82.9%), correctly detecting 554 of 692 (FN: 138) ELISA-positive samples. Specificity was 89.4% (95% CI: 86.3–91.9%), with 49 false positives among 464 ELISA-negative samples. Overall accuracy reached 83.8% (95% CI: 81.6–85.8%), corresponding to good agreement with ELISA (κ = 0.67) (Table 4). One hundred forty-three samples were not included in the sensitivity and specificity calculation because they presented indeterminate results in the ELISA test.

**Table 4:** Overall performance of the IgG-RDT using plasma samples from Rondônia, Brazil.

Parameters	Value (95% CI)
Sensitivity	80.1% (76.9 – 82.9)
Specificity	89.4% (86.3 – 91.9)
Accuracy	83.8% (81.6 – 85.8)
*Kappa* index	0.67 (good agreement)

Global sensitivity, specificity, accuracy, and kappa index were calculated from all samples tested in endemic areas (Candeias do Jamari and Porto Velho).

## Discussion

Despite efforts to eradicate malaria, *Plasmodium vivax* remains a significant challenge, particularly in regions moving toward elimination, due to its widespread prevalence and complex epidemiology [29]. The aims of elimination proposed by the National Malaria Elimination Plan, require an optimization of diagnostic tools in Brazil, since commercially available tests are not sufficient to accomplish the requirements of elimination, control, and monitoring of malaria transmission [1].

It is well established that *P. vivax* blood-stage infections induce robust IgG antibody responses against a wide range of *P. vivax* proteins, even after low-density asymptomatic infections [30]. Given the limitations of microscopy, molecular tests, and antigen capture RDTs, especially in detecting individuals with low parasitemia or those who may harbor hypnozoites from *P. vivax* infection [5,31], a rapid antibody-based test for *P. vivax* antigens emerges as a promising alternative. Such a tool could help fill the gaps in detecting asymptomatic or recently exposed individuals and expand diagnostic access in remote areas of the country, where malaria incidence remains high.

Several studies have demonstrated that individuals with chronic exposure and multiple malaria episodes tend to produce higher levels of antigen-specific antibodies [24,32]. By using a standardization panel, we found that, among asymptomatic individuals, false-negative cases (5/49) occurred in samples with low ELISA reactivity indices, corresponding to individuals who had reported not been infected previously for more than one year and exhibited antibody titers near the cut-off. These findings may suggest early asymptomatic infections in which antibody responses were still developing, particularly considering that prior exposure was not recent and that these individuals remained asymptomatic for at least 45 days after sample collection.

Despite the false negatives, the rapid test successfully detected most infected individuals, yielding promising sensitivity and specificity results demonstrating its potential as a practical tool for detecting and monitoring recent exposure and active infection. The one-year longitudinal study (starting from day 28 after treatment) indicated a gradual decrease in antibody levels post-treatment with chloroquine plus primaquine, with higher reactivity within the first six months. This pattern reflects the natural decline in antibody levels after treatment and suggests that a positive result on the ELISA is associated with a recent infection [14,33,34], with approximately 96% probability of parasite exposure within the preceding nine months. Thus, the developed Ab-RDT showed potential for estimating the temporal window of exposure, which is highly relevant for transmission surveillance, providing information about recent infection or risk of harboring the parasite [35–37].

In Porto Velho, 65.3% of recurrences are reinfections (recurrence after 60 days after treatment), while relapses (recurrence between 28–60 days after treatment) comprise 29.5% of recurrences [38]. In this scenario, a positive result in the Ab-RDT after 9 months of treatment might indicate an asymptomatic infection or frequent exposure to the parasite. In both cases, the use of an Ab-RDT is informative and supports the deployment of personalized strategies, including molecular diagnosis of subpatent infection, a new round of treatment based on presumptive diagnosis, and/or household control measures, such as insecticides, bed nets, and repellents. Within this scenario, the Ab-RDT could identify hotspots of transmission and reservoirs in cross-sectional assessments, 9–12 months apart, guiding control strategies to a new level. The Ab-RDT could also identify areas almost free of transmission, where control measures might be directed to individuals with mobility to endemic areas, serving as a reliable, practical, and affordable tool for sub-national certification of malaria eradication.

Our second stage cross-sectional study yielded results comparable to those obtained during test standardization. The test successfully identified individuals exposed to the parasite, even among asymptomatic participants. Although some false negatives occurred, the Ab-RDT detected several samples with reactivity indexes near the ELISA cut-off. Furthermore, it maintained high specificity, wherein all negative reference samples were also negative on the RDT, an essential feature for epidemiological surveillance applications [39].

In the longitudinal study (third validation stage), heterogeneity in serological responses among qPCR-positive individuals reflected the complexity of *P. vivax* immunity. IgG-negative cases in qPCR-confirmed infections may indicate asymptomatic primary infections with low parasitemia or a lack of immune memory against the selected antigens in individuals with a prior infection history. Some individuals presented a single qPCR-positive sample, possibly indicating early reinfection or recurrence, or even unsuccessful infections, for which unknown reasons the parasite could not establish a productive infection. Another explanation is false-positive qPCR results, which are inherent to the methodology. Although all the controls performed as expected, it cannot be ruled out that point contamination could happen in some wells. For instance, we consider a participant positive if they have at least one positive result in triplicate, even if the *Cq*s is higher than 35, which is very sensitive to false positives. Nonetheless, oscillating asymptomatic detection patterns were also observed, consistent with previous findings [5].

Individuals negative by qPCR but showing weak RDT signals and/or low ELISA reactivity indices may fit three hypotheses: first, IgG antibody titers may be declining after an extended period without parasite re-exposure; second, regarding the Ab-RDT, technical limitations may arise from the sample type used, since switching from plasma to whole blood can introduce blood-related interferences, such as variations in viscosity and hemoglobin concentration, which can affect fluid migration and signal intensity; third, cross-reactivity with other pathogens, as the region is endemic for other diseases.

Nonetheless, the high agreement between Ab-RDT and ELISA, consistently observed over 5 weeks, suggests that the rapid test accurately reflects the population’s serological profile.

The significant association between positive results on both tests and subsequent qPCR-detectable infection (HR > 3 in both assays) indicates that the presence of antibodies against *Pv*MSP1₁₉ and *Pv*AMA-1 antigens not only reflects recent exposure but also anticipates a significantly higher risk of active infection in the following weeks. This finding indicates that, although qPCR fluctuates and fails to detect asymptomatic infections at certain points, the Ab-RDT is a stable marker over time and might indicate a silent infection that can be confirmed with other highly sensitive methodologies. One suitable strategy is to use Ab-RDT as a screening method, directing only positive individuals for further diagnosis and reducing the costs and effort of testing the entire population. This approach is particularly relevant given the limitations of traditional strategies (MDA: mass drug administration; and MSAT: mass screening and treatment), which end up treating many people unnecessarily or rely on methods that only detect active bloodstream infections, failing to identify asymptomatic carriers with low parasitemia or individuals carrying hypnozoites. In this context, the Ab-RDT could also be integrated into active case detection campaigns, allowing for more precise selection of individuals for serological Test-And-Treat (SeroTAT) strategy, prioritizing those at higher risk of infection [6,11,40].

The development of a diagnostic test that meets the Ministry’s requirements to support malaria elimination strategies has become essential, particularly given the existing gaps in current diagnostics [1,41]. A study by Longley et al. [9] identified a panel of 8 proteins that, together, achieved 80% sensitivity and specificity for identifying recent *P. vivax* infections when analyzing individuals who had PCR-detectable blood-stage *P. vivax* infections within the 9 months before measurement of antibody responses. Although combining proteins improves classification accuracy for infections within the previous nine months [42], adding multiple proteins substantially increases test production costs. The proposed rapid test achieved overall sensitivity and specificity of 80.1% and 89.4%, respectively, using only two parasite antigens, reflecting advantages primarily in terms of production cost.

Our study has some limitations that must be acknowledged. First, the Ab-RDT is not designed to detect active infection. Therefore, it is not suitable as a point-of-care tool for individual diagnosis, but rather as a tool for epidemiological monitoring to support decision-making in elimination scenarios, where both parasites and serological titers are residual. We used a very sensitive qPCR protocol, and our definition of a positive qPCR case can be affected by false-positive results, although we followed all the good practices during sample processing and qPCR. It is important to notice that we previously showed that even individuals with inconsistent and negative qPCR results were able to infect mosquitoes [5,23]. Therefore, new studies are necessary with a greater sample size to validate the findings shown here. Due to the study design and the availability of epidemiological data, it was not possible to comprehensively control all potential confounders in the present analysis. Factors such as age, cumulative malaria exposure, time since previous infection, host immune background, and variability in antibody acquisition and persistence may all influence IgG levels against *Pv*MSP1_19_ and *Pv*AMA1. Therefore, serological responses may differ substantially between individuals, potentially affecting Ab-RDT sensitivity and the interpretation of results, particularly in low-transmission or heterogeneous endemic settings. Nevertheless, participants were recruited from the same endemic area and were subjected to the same inclusion criteria, which likely reduced part of the heterogeneity related to malaria exposure. In addition, this study was designed to evaluate the diagnostic performance of the assay under real-world field conditions rather than to investigate determinants of immune response variability.

Despite these limitations, the test may still have practical utility in real-world settings where malaria control measures have substantially reduced the incidence of *P. vivax* infection. In such scenarios, exposed individuals could be rapidly identified using the Ab-RDT and promptly referred for confirmatory testing and treatment, reducing operational costs and improving the efficiency of targeted malaria control interventions. Importantly, serology-guided screening strategies have previously been incorporated into malaria elimination campaigns in Brazil, where they contributed to the identification and treatment of exposed individuals in low-transmission settings. The historical elimination campaign conducted in Santa Catarina between 1980 and 1985 demonstrated the feasibility of integrating serological screening with active case detection and treatment strategies as part of a broader malaria elimination effort [12].

The results obtained in this study reinforce the potential of the Ab-RDT as a supportive tool for surveillance of *P. vivax* malaria, particularly by enabling the use of whole blood samples, which facilitates its application in remote areas. It is important to emphasize that the rapid antibody detection test is intended mainly to aid in monitoring transmission in regions moving towards eradication, thereby enabling more strategic malaria control efforts.

## Supporting information

S1 Raw Data Anonymized dataset. (XLSX)
